# Can NO Signaling and Its Metabolism Be Used to Improve Nutrient Use Efficiency? Toward a Research Agenda

**DOI:** 10.3389/fpls.2022.787594

**Published:** 2022-02-15

**Authors:** Agustina Buet, Melisa Luquet, Guillermo E. Santa-María, Andrea Galatro

**Affiliations:** ^1^Centro de Investigaciones en Toxicología Ambiental y Agrobiotecnología del Comahue (CITAAC), Consejo Nacional de Investigaciones Científicas y Técnicas (CONICET)-Universidad Nacional del Comahue, subsede Instituto de Biotecnología Agropecuaria del Comahue (IBAC), Cinco Saltos, Argentina; ^2^Facultad de Ciencias Agrarias y Forestales (FCAyF), Universidad Nacional de La Plata (UNLP), La Plata, Argentina; ^3^Instituto de Fisiología Vegetal (INFIVE), Universidad Nacional de La Plata (UNLP)-Consejo Nacional de Investigaciones Científicas y Técnicas (CONICET), La Plata, Argentina; ^4^Instituto Tecnológico Chascomús (INTECH), Universidad Nacional de San Martín (UNSAM)-Consejo Nacional de Investigaciones Científicas y Técnicas (CONICET), Chascomús, Argentina

**Keywords:** nutrient use efficiency, Nitric Oxide, nitrogen, phosphorus, potassium, acquisition, utilization

## Introduction

One important issue to be faced by modern agriculture is ensuring an efficient use of the essential nutrients taken up from the soil by crops, thus helping to reduce the economic costs and by-side environmental effects derived from addition of fertilizers, which -frequently- involve the use of non-renewable resources. Improving use efficiency of the major nutrients contained in these fertilizers, nitrogen (N), phosphorus (P) and potassium (K), can be afforded through multiple strategies and must be thought in concert with the prevalence of a panoply of biotic and abiotic stresses. Therefore, simultaneous attention must be paid to the signaling network involved in setting Nutrient Use Efficiency (NUE) and to the acclimation of plants to a wide range of environments.

Nitric Oxide (NO) has been shown to influence some aspects of N, P and K nutrition as well as the response of plants to several stress conditions (Buet et al., 2019; Kolbert et al., 2019). The possibility to use NO metabolism/signaling to improve NUE has been recently contemplated (Del Castello et al., 2020). However, there is considerable uncertainty regarding how NO manipulation can be readily used to improve NUE. In this opinion article, we highlight the unknowns that must be known in order to make that use feasible while proposing some priorities for a research agenda. Given the various roles covered by NO in plants, special attention should be paid to the occurrence of unwanted collateral effects derived from NO manipulation. We emphasize that the use of NO to improve NUE will require deep knowledge on the signaling network involved, as well as a suitable quantitative assessment of NUE and its components.

## NUE: Components and Mechanisms

NUE is an increasingly used concept that refers to the yield, or dry matter accumulation, relative to the availability of a given nutrient in the soil solution. It can be decomposed into Nutrient Acquisition Efficiency (NAE) and Nutrient Utilization Efficiency (NUtE). NAE informs on the amount of nutrient captured by plants relative to nutrient availability in the soil, while NUtE informs on the yield (or dry matter accumulated) per unit of nutrient incorporated (Rose and Wissuwa, 2012; Santa-María et al., 2015).

Several plant traits contribute to determining NAE; including the root growth pattern, the symbiotic and non-symbiotic relationships with soil microorganisms, the release of compounds that modify nutrient availability, and the activity of the transporters in the root absorbing zone. Current knowledge indicates that NUtE involves traits related to the distribution of nutrients and dry matter among different organs, the capacity to modify the metabolism, and the partial substitution of an element by a related one. These traits could involve several mechanisms, extensively revised for N, P and K (Veneklaas et al., 2012; Cormier et al., 2016; White et al., 2021).

## NUE: The Need to Quantify the Impact of NO

Available information indicates that NO influences many processes underlying traits related to NUE. As an example, it has been shown that, under conditions of variable N supply, root growth, N uptake and assimilation are influenced by NO (Frungillo et al., 2014; Sun et al., 2015; Balotf et al., 2018). Reciprocally, NO is coupled with N metabolism, as NO can be generated from nitrite, arising from N assimilation, and from arginine arising from N metabolism. Moreover, NO generation may be affected by the forms (${\text{NO}}_{3}^{-}$ or ${\text{NH}}_{4}^{+}$) and the levels of N supply (Buet et al., 2019), potentially influencing NUE. Evidence also supports a role of NO on key responses to low P supply (Wang et al., 2010; Zhu et al., 2017; Ramos-Artuso et al., 2018), and in K nutrition (Chen et al., 2016). In addition, proteins likely involved in setting NUE are subjected to NO post-translational modifications such as occur with the P-transporter PHT3;1 (Fares et al., 2011), and -knowing the cysteine residues involved- glutamine synthetase (Silva et al., 2019) and S-nitrosoglutathione Reductase 1, GSNOR1 (Frungillo et al., 2014; Guerra et al., 2016). NO can also affect the expression of NUE-likely-related transcripts, such as those coding for nitrate reductase, other enzymes of the N assimilation pathway (Balotf et al., 2018), and the P-transporter *OsPT2* (Zhu et al., 2017).

Despite the evidence indicating that NO influences mechanisms underlying nutrient acquisition and utilization, there are scarce measurements of NAE and NUtE in the framework of NO research (e.g., Del Castello et al., 2021; Gautam et al., 2021). Thus, the precise quantitative influence of NO on them remains mostly unknown. This should prompt to perform measurements of these efficiencies, an aspect that needs the use of adequate phenotyping protocols. In this regard, NAE must be preferentially evaluated with plants grown in soil, as interactions with soil matrix and microorganisms could be relevant. Measurements of NUtE must be preferentially performed with growth systems that help to avoid large disparities in nutrient accumulation (Rose and Wissuwa, 2012), accompanied by the use of appropriate estimators (Santa-María et al., 2015). Performing measurements of NAE and NUtE with validated phenotyping protocols in the context of NO research constitutes the first item of our research agenda.

## NUE Improvement: The Need to Know the Balance of Endogenous NO Levels and NO Signaling Networks

The impact of NO signaling and metabolism to improve NUE will depend on the way by which endogenous NO levels and their subsequent actions are set in a particular environment. This is a complex and essentially unknown issue for most nutritional conditions and, as described for several processes, likely involves interactions with other signals such as reactive oxygen species and hormones (Kolbert et al., 2019). It should be noticed that NO may exert its actions in a compartmentalized way, involving a delicate equilibrium between NO generation and consumption, which could be critical to set a given biological outcome (Hancock, 2019). The precise contribution of NO generation and consumption pathways to determine NO levels under growth conditions compatible with adequate measurements of NAE and NUtE remains essentially unknown and constitutes a second component of the research agenda. The relevance of those pathways in setting NUE should not be underestimated as exemplified by the effect of variable nitrate supply on maize root growth, where NO production and scavenging, associated with a coordinated spatiotemporal expression of nitrate reductase and non-symbiotic hemoglobins (involved in NO generation and consumption, respectively), finally contribute to defining primary root growth (Trevisan et al., 2011; Manoli et al., 2014). Noticeably, the effects displayed by NO have been shown to depend not only on the levels of nutrient supply, but also on the interaction with other environmental conditions (Du et al., 2016).

NUE relates the economy of mineral elements and carbon. Maintenance of NO generation within a narrow range is necessary to optimize growth (Sánchez-Vicente et al., 2019) while less is known about the effect of NO balance on the control of the elemental composition (Babasheikhali et al., 2020; Sohag et al., 2020). The addition of NO donors, as well as the use of mutants or transgenic plants displaying enhanced NO production in several tissues, would lead to NO imbalances, followed by a cascade of NO actions that finally are integrated into a whole plant outcome. The occurrence of multiple NO induced events is indicated by transcriptome studies unveiling that exogenous NO affects the expression of many transcription factors (Hussain et al., 2016; Imran et al., 2018), and can also influence the activity of some of them as shown by *in vitro* studies (Serpa et al., 2007; Tavares et al., 2014). Besides, work with the *nox1* mutant -displaying enhanced NO production-disclosed relevant modifications in the proteome (Hu et al., 2014). In this context, it should be considered that low levels of different nutrients—or other stress conditions—could coexist, involving an interplay among signaling cascades. Therefore, particularly following exogenous NO treatments, the possibility of affecting more than a single signaling cascade exerting non-predictable impacts on traits contributing to different stress conditions should be contemplated. This can be partially illustrated, considering that the addition of certain NO donors could lead—in some plants, under particular growth conditions—to enhanced N, P and/or K uptake (Sun et al., 2015; Ramos-Artuso et al., 2018; Alnusairi et al., 2021). However, in some environments these NO donors may negatively impact on the main systems determining K influx (Xia et al., 2014; Oliferuk et al., 2020). On the other hand, abiotic stresses like salinity and cold, hamper the nutrition of some elements, and addition of NO could help plants to reduce detrimental effects exerted on growth and the accumulation of certain nutrients (Sohag et al., 2020; Alnusairi et al., 2021). Therefore, in deep studies on NO signaling cascades (and specially their connections) for interacting levels of nutrient supply and stress conditions should be reinforced in the research agenda.

Having in mind all the issues just discussed, it seems unlikely that NO donors can mirror the specific acclimation roles of endogenous NO during nutrient deficiencies, and interactive no obvious effects on NUE for different elements may be expected. Nevertheless, the possibility to use NO-releasing nanoparticles should be not excluded as they may deliver NO in a more controlled way (as compared to free forms), promoting growth under particular stress conditions (Silveira et al., 2021). Similar considerations may apply for specific transgenic plants. Considering the issues here mentioned, another aspect to be incorporated into the research agenda is the need to provide an integral evaluation of the plant performance, especially its stability across environments, for any NO manipulation approach.

## Future Directions

**Figure 1:**
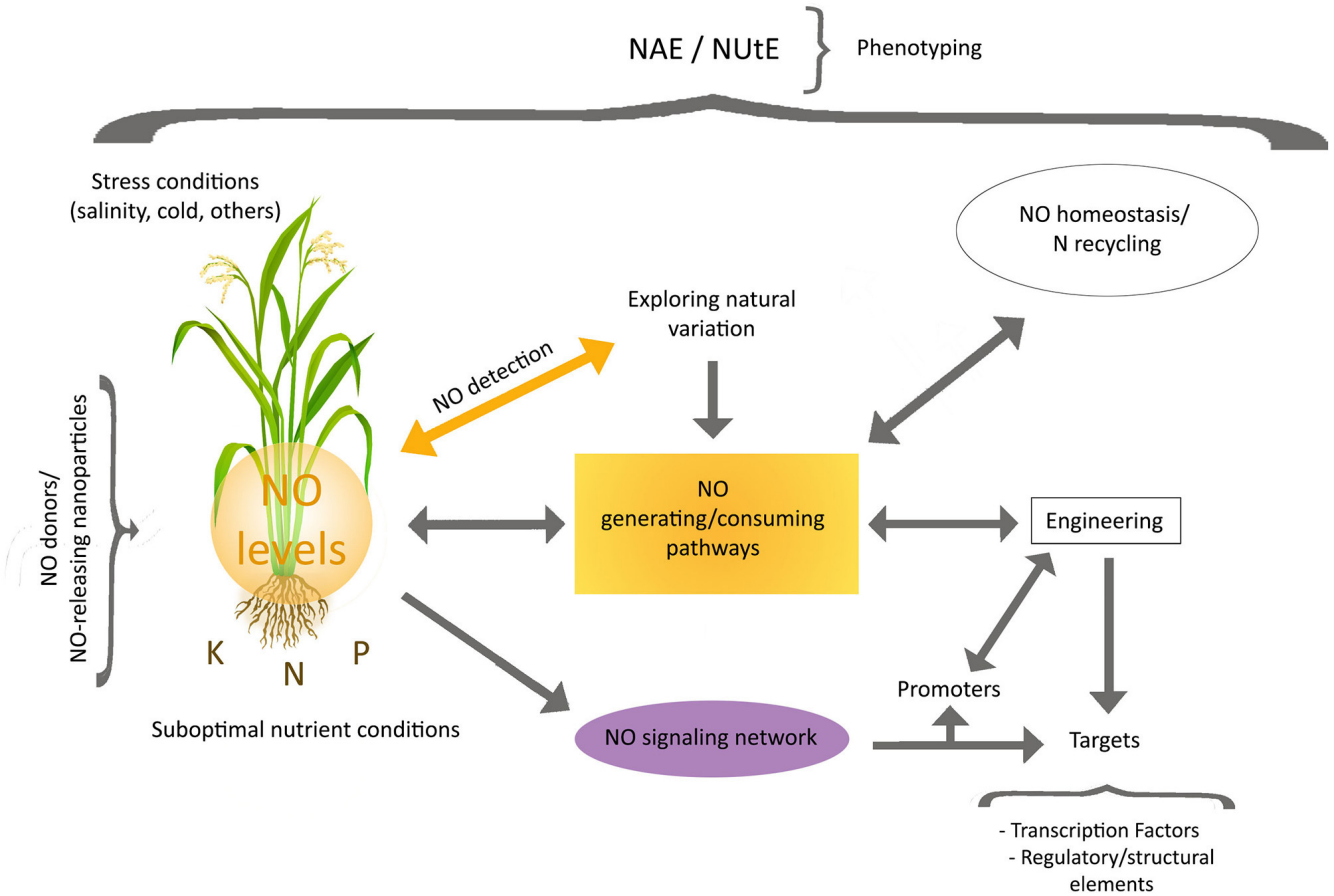
Toward the use of NO signaling and metabolism for NUE improvement. Future research directions should include the dissection of the routes controlling local NO levels and NO signaling associated with suboptimal nutrient supply. Studies focusing on natural variation in NO balance and signaling, as well as adequate measurements of NUtE and NAE, will be needed. Engineering of localized NO signals (through NO generating/consuming proteins), NO homeostasis and associated N recycling as well as NO targets, will be also necessary for the development of potential strategies leading to NUE enhancement while reducing possible by-side effects. The identification of transcription factors and structural proteins acting in the NO signaling network, under suboptimal specific nutrient stress conditions, will provide additional tools for the development of new engineering procedures. Stress conditions hampering growth and nutrient homeostasis should be also taken into consideration. The precise impact of strategies based on the use of NO donors/NO-releasing particles must be evaluated.

In the context of NUE improvement, studies on the endogenous NO signaling pathways operating under conditions of suboptimal nutrient supply will be relevant, as they may disclose the targets of NO action, which should be complemented by the identification of the routes leading to set local NO levels (Figure 1). The subsequent engineering of localized NO signals, as well as of the NO homeostasis and associated N recycling (Del Castello et al., 2020), along with that of NO targets, could be the base of future strategies for NUE enhancement. A strategy based on alteration of localized NO levels will need innovative technical procedures to estimate them, including the use of genetically encoded sensors (Safavi-Rizi, 2021). The alteration of NO levels could be achieved by the introgression of NO generating/consuming proteins under the control of well-suited promoters, acting under specific nutritional conditions, thus reducing the by-side effects emerging from uncontrolled NO use. Introgression of proteins affecting NO balance, may also result in N metabolism modifications, as shown in Arabidopsis plants expressing *SyNOS*, from *Sinechococcus*, which besides to generate NO from arginine, mediates its conversion to nitrate leading to an enhancement of NUE under low N supply (Del Castello et al., 2021). In addition, identification of transcription factors and structural proteins acting downstream in the NO signaling network, elicited by specific nutrient stress conditions, may be used for a wide range of approaches to express or edit proteins contributing to traits for enhanced NAE and/or NUtE. All these items must be also included in the research agenda here outlined.

Furthermore, screening of large collections, under variable nutrient supply conditions, using either mutagenized plants or high-diversity panels, may help to identify key elements involved in NO generation and signaling yet unknown. The use of the first procedure led to uncover the role of pyridoxal in the control of K transport (Xia et al., 2014). Regarding the second one, a recent genome-wide association study using a panel of genotyped barley accessions uncovered several marker-associations with NO release (Nagel et al., 2019). To our knowledge no studies have been conducted to disclose the existence of natural variation in NO release for crop plants deprived of major nutrients and to examine its potential association with NUE variation among genotypes. This is an additional issue to be afforded by future research.

In conclusion, the extent to what and how NO signaling/metabolism can be manipulated to impact on NUE improvement will greatly depend on the accomplishment of a detailed research agenda just outlined here (Figure 1). This will require careful experimental designs and the use of suitable protocols of phenotyping across multiple environments.

## Author Contributions

AB, ML, GS-M, and AG contributed to writing the draft manuscript and editing. All authors approved the submitted version.

## Funding

This work was supported by funds from Agencia Nacional de Promoción Científica y Tecnológica (ANPCyT) [PICT 2017-2492 to AG and PICT 2019-03103 to GS-M] and from Consejo Nacional de Investigaciones Científicas y Técnicas (CONICET) [PIP 2017-2019 and 11220170100629CO].

## Conflict of Interest

The authors declare that the research was conducted in the absence of any commercial or financial relationships that could be construed as a potential conflict of interest.

## Publisher's Note

All claims expressed in this article are solely those of the authors and do not necessarily represent those of their affiliated organizations, or those of the publisher, the editors and the reviewers. Any product that may be evaluated in this article, or claim that may be made by its manufacturer, is not guaranteed or endorsed by the publisher.

## References

[B1] Alnusairi G. S. Mazrou Y. S. Qari S. H. Elkelish A. A. Soliman M. H. Eweis M.. (2021). Exogenous nitric oxide reinforces photosynthetic efficiency, osmolyte, mineral uptake, antioxidant, expression of stress-responsive genes and ameliorates the effects of salinity stress in wheat. Plants 10, 1693. 10.3390/plants10081693 34451738 PMC8400961

[B2] Babasheikhali M. M. Jabbarzadeh Z. Amiri J. Barin M. (2020). Impact of salicylic acid and nitric oxide on improving growth and nutrients uptake of rose in alkaline soil conditions. J. Plant Nutr. 43, 667–681. 10.1080/01904167.2019.1701023

[B3] Balotf S. Islam S. Kavoosi G. Kholdebarin B. Juhasz A. Ma W. (2018). How exogenous nitric oxide regulates nitrogen assimilation in wheat seedlings under different nitrogen sources and levels. PLoS ONE 13, e0190269. 10.1371/journal.pone.0190269 29320529 PMC5761883

[B4] Buet A. Galatro A. Ramos-Artuso F. Simontacchi M. (2019). Nitric oxide and plant mineral nutrition: current knowledge. J. Exp. Bot. 70, 4461–4476. 10.1093/jxb/erz129 30903155

[B5] Chen Z. H. Wang Y. Wang J. W. Babla M. Zhao C. García-Mata C.. (2016). Nitrate reductase mutation alters potassium nutrition as well as nitric oxide-mediated control of guard cell ion channels in *Arabidopsis*. New Phytol. 209, 1456–1469. 10.1111/nph.13714 26508536

[B6] Cormier F. Foulkes J. Hirel B. Gouache D. Moënne-Loccoz Y. Le Gouis J. (2016). Breeding for increased nitrogen-use efficiency: a review for wheat (*T. aestivum L*.). Plant Breed 135, 255–278. 10.1111/pbr.12371

[B7] Del Castello F. Foresi N. Nejamkin A. Lindermayr C. Buegger F. Lamattina L.. (2021). Cyanobacterial NOS expression improves nitrogen use efficiency, nitrogen-deficiency tolerance and yield in *Arabidopsis*. Plant Sci. 307, 110860. 10.1016/j.plantsci.2021.110860 33902845

[B8] Del Castello F. Nejamkin A. Foresi N. Lamattina L. Correa-Aragunde N. (2020). Chimera of globin/nitric oxide synthase: toward improving nitric oxide homeostasis and nitrogen recycling and availability. Front. Plant Sci. 11, 1461. 10.3389/fpls.2020.575651 33101345 PMC7554344

[B9] Du S. Zhang R. Zhang P. Liu H. Yan M. Chen N.. (2016). Elevated CO_2_-induced production of nitric oxide (NO) by NO synthase differentially affects nitrate reductase activity in *Arabidopsis* plants under different nitrate supplies. J. Exp. Bot. 67, 893–904. 10.1093/jxb/erv506 26608644

[B10] Fares A. Rossignol M. Peltier J. B. (2011). Proteomics investigation of endogenous S-nitrosylation in *Arabidopsis*. Biochem. Biophys. Res. Commun. 416, 331–336. 10.1016/j.bbrc.2011.11.036 22115780

[B11] Frungillo L. Skelly M. J. Loake G. J. Spoel S. H. Salgado I. (2014). S-nitrosothiols regulate nitric oxide production and storage in plants through the nitrogen assimilation pathway. Nat. Commun. 5, 5401. 10.1038/ncomms6401 25384398 PMC4229994

[B12] Gautam H. Sehar Z. Rehman M. T. Hussain A. AlAjmi M. F. Khan N. A. (2021). Nitric oxide enhances photosynthetic nitrogen and sulfur-use efficiency and activity of ascorbate-glutathione cycle to reduce high temperature stress-induced oxidative stress in rice (*Oryza sativa* L.) plants. Biomolecules 11, 305. 10.3390/biom11020305 33670537 PMC7922496

[B13] Guerra D. Ballard K. Truebridge I. Vierling E. (2016). S-nitrosation of conserved cysteines modulates activity and stability of S-nitrosoglutathione reductase (GSNOR). Biochemistry 55, 2452–2464. 10.1021/acs.biochem.5b01373 27064847 PMC4974627

[B14] Hancock J. T.. (2019). Considerations of the importance of redox state for reactive nitrogen species action. J. Exp. Bot. 70, 4323–4331. 10.1093/jxb/erz067 30793204

[B15] Hu W.-J. Chen J. Liu T.-W. Liu X. Chen J. Wu F.-H.. (2014). Comparative proteomic analysis on wild type and nitric oxide-overproducing mutant (*nox1*) of *Arabidopsis thaliana*. Nitric Oxide 36, 19–30. 10.1016/j.niox.2013.10.008 24184441

[B16] Hussain A. Mun B. G. Imran Q. M. Lee S. U. Adamu T. A. Shahid M.. (2016). Nitric Oxide mediated transcriptome profiling reveals activation of multiple regulatory pathways in *Arabidopsis thaliana*. Front. Plant Sci. 7, 975. 10.3389/fpls.2016.00975 27446194 PMC4926318

[B17] Imran Q. M. Hussain A. Lee S. U. Mun B. G. Falak N. Loake G. J.. (2018). Transcriptome profile of NO-induced Arabidopsis transcription factor genes suggests their putative regulatory role in multiple biological processes. Sci. Rep. 8, 771. 10.1038/s41598-017-18850-5 29335449 PMC5768701

[B18] Kolbert Z. Barroso J. B. Brouquisse R. Corpas F. J. Gupta K. J. Lindermayr C.. (2019). A forty year journey: the generation and roles of NO in plants. Nitric Oxide 93, 53–70. 10.1016/j.niox.2019.09.006 31541734

[B19] Manoli A. Begheldo M. Genre A. Lanfranco L. Trevisan S. Quaggiotti S. (2014). NO homeostasis is a key regulator of early nitrate perception and root elongation in maize. J. Exp. Bot. 65, 185–200. 10.1093/jxb/ert358 24220653 PMC3883287

[B20] Nagel M. Alqudah A. M. Bailly M. Rajjou L. Pistrick S. Matzig G.. (2019). Novel loci and a role for nitric oxide for seed dormancy and preharvest sprouting in barley. Plant Cell Environ. 42, 1318–1327. 10.1111/pce.13483 30652319

[B21] Oliferuk S. Simontacchi M. Rubio F. Santa-María G. E. (2020). Exposure to a natural nitric oxide donor negatively affects the potential influx of rubidium in potassium-starved Arabidopsis plants. Plant Physiol. Biochem. 150, 204–208. 10.1016/j.plaphy.2020.02.043 32155448

[B22] Ramos-Artuso F. Galatro A. Buet A. Santa-María G. E. Simontacchi M. (2018). Key acclimation responses to phosphorus deficiency in maize plants are influenced by exogenous nitric oxide. J. Plant Physiol. 222, 51–58. 10.1016/j.jplph.2018.01.001 29407549

[B23] Rose T. J. Wissuwa M. (2012). Rethinking internal phosphorus utilization efficiency: a new approach is needed to improve PUE in grain crops, in advances in agronomy. Adv. Agron. 116, 185–217. 10.1016/B978-0-12-394277-7.00005-1

[B24] Safavi-Rizi V.. (2021). Towards genetically encoded sensors for nitric oxide bioimaging in planta. Plant Physiol. 187, 477–479. 10.1093/plphys/kiab232 34608950 PMC8491015

[B25] Sánchez-Vicente I. Fernández-Espinosa M. G. Lorenzo O. (2019). Nitric oxide molecular targets: reprogramming plant development upon stress. J. Exp. Bot. 70, 4441–4460. 10.1093/jxb/erz339 31327004 PMC6736187

[B26] Santa-María G. E. Moriconi J. I. Oliferuk S. (2015). Internal efficiency of nutrient utilization: what is it and how to measure it during vegetative plant growth? J. Exp. Bot. 66, 3011–3018. 10.1093/jxb/erv162 25922492

[B27] Serpa V. Vernal J. Lamattina L. Grotewold E. Cassia R. Terenzi H. (2007). Inhibition of AtMYB2 DNA-binding by nitric oxide involves cysteine S-nitrosylation. Biochem. Biophys. Res. Commun. 361, 1048–1053. 10.1016/j.bbrc.2007.07.133 17686455

[B28] Silva L. S. Alves M. Q. Seabra A. R. Carvalho H. G. (2019). Characterization of plant glutamine synthetase S-nitrosation. Nitric Oxide 88, 73–86. 10.1016/j.niox.2019.04.006 31026500

[B29] Silveira N. M. Prataviera P. J. Pieretti J. C. Seabra A. B. Almeida R. L. Machado E. C.. (2021). Chitosan-encapsulated nitric oxide donors enhance physiological recovery of sugarcane plants after water deficit. Environ Exp Bot 190, 104593. 10.1016/j.envexpbot.2021.104593

[B30] Sohag A. A. M. Tahjib-Ul-Arif M. Afrin S. Khan M. K. Hannan M. A. Skalicky M.. (2020). Insights into nitric oxide-mediated water balance, antioxidant defence and mineral homeostasis in rice (*Oryza sativa* L.) under chilling stress. Nitric Oxide 100, 7–16. 10.1016/j.niox.2020.04.001 32283262

[B31] Sun H. Li J. Song W. Tao J. Huang S. Chen S.. (2015). Nitric oxide generated by nitrate reductase increases nitrogen uptake capacity by inducing lateral root formation and inorganic nitrogen uptake under partial nitrate nutrition in rice. J. Exp. Bot. 66, 2449–2459. 10.1093/jxb/erv030 25784715 PMC4986861

[B32] Tavares C. P. Vernal J. Delena R. A. Lamattina L. Cassia R. Terenzi H. (2014). S-nitrosylation influences the structure and DNA binding activity of AtMYB30 transcription factor from *Arabidopsis thaliana*. Biochim. Biophys. Acta Proteins Proteom. 1844, 810–817. 10.1016/j.bbapap.2014.02.015 24583075

[B33] Trevisan S. Manoli A. Begheldo M. Nonis A. Enna M. Vaccaro S.. (2011). Transcriptome analysis reveals coordinated spatiotemporal regulation of hemoglobin and nitrate reductase in response to nitrate in maize roots. New Phytol. 192, 338–352. 10.1111/j.1469-8137.2011.03822.x 21762167

[B34] Veneklaas E. J. Lambers H. Bragg J. Finnegan P. M. Lovelock C. E. Plaxton W. J.. (2012). Opportunities for improving phosphorus-use efficiency in crop plants. New phytol. 195, 306–320. 10.1111/j.1469-8137.2012.04190.x 22691045

[B35] Wang B. L. Tang X. Y. Cheng L. Y. Zhang A. Z. Zhang W. H. Zhang F. S.. (2010). Nitric oxide is involved in phosphorus deficiency-induced cluster-root development and citrate exudation in white lupin. New Phytol. 187, 1112–1123. 10.1111/j.1469-8137.2010.03323.x 20553395

[B36] White P. J. Bell M. J. Djalovic I. Hinsinger P. Rengel Z. (2021). Potassium use efficiency of plants, in Improving Potassium Recommendations for Agricultural Crops, eds Murrell T. S. Mikkelsen R. L. Sulewski G. Norton R. Thompson M. L. (Cham: Springer Nature Switzerland AG), 119–145. 10.1007/978-3-030-59197-7_5

[B37] Xia J. Kong D. Xue S. Tian W. Li N. Bao F.. (2014). Nitric oxide negatively regulates AKT1-mediated potassium uptake through modulating vitamin B6 homeostasis in *Arabidopsis*. Proc. Natl. Acad. Sci. U.S.A. 111, 16196–16201. 10.1073/pnas.1417473111 25355908 PMC4234564

[B38] Zhu X. F. Zhu C. Q. Wang C. Dong X. Y. Shen R. F. (2017). Nitric oxide acts upstream of ethylene in cell wall phosphorus reutilization in phosphorus deficient rice. J. Exp. Bot. 68, 753–760. 10.1093/jxb/erw480 28064177 PMC6055659

